# Upregulation of ubiquitin conjugating enzyme E2B (Ube2b) ameliorates neuropathic pain by regulating Kcna2 (potassium voltage-gated channel subfamily A member 2) in primary afferent neurons

**DOI:** 10.1080/21655979.2021.1976895

**Published:** 2021-10-10

**Authors:** Yuanzhi Peng, Qingqing Zhang, Hao Cheng, Guizhen Yan, Chunli Xing

**Affiliations:** a Department of Anesthesiology and SICU, Xinhua Hospital, School of Medicine, Shanghai Jiao Tong University, Shanghai,China; b Department of Cardiovascular Surgery, Changhai Hospital, the Navy Medical University, Shanghai, China; c Department of Neurology, People’s Hospital of Lixia District of Jinan, Jinan, Shandong, China

**Keywords:** Ube2b, DNMT3a, Kcna2, neuropathic pain

## Abstract

Neuropathic pain is a kind of pain caused by damage to somatosensory nervous system. Currently, neuropathic pain is still a medical problem for clinicians. Ubiquitin conjugating enzyme E2B (Ube2b) is validated to be implicated with nerve function, but whether Ube2b can play a role in neuropathic pain is still elusive. In this work, we constructed chronic constriction injury (CCI) rat model by ligating the left sciatic nerve, Ube2b protein expression was confirmed to be decreased in spinal cord tissues of CCI rats via Western blot analysis and immunofluorescence (IF) staining. Moreover, Ube2b elevation alleviated the thermal hyperalgesia and mechanical hyperalgesia in CCI rats according to paw withdrawal thermal latency (PWTL) and paw withdrawal mechanic threshold (PWMT). In addition, Hematoxylin-eosin staining revealed that Ube2b elevation suppressed chronic sciatic nerve injury. All these data suggested that Ube2b could ameliorate neuropathic pain in CCI rats. Mechanically, Ube2b upregulation elevated the protein level of Kcna2 (potassium voltage-gated channel subfamily A member 2) and decreased the protein level of DNMT3a (DNA methyltransferase 3 alpha). Ube2b elevation could increase Kcna2 expression via suppressing DNMT3a. Rescue assays unveiled that Ube2b overexpression modulated-mechanical hyperalgesia and thermal hyperalgesia were reversed by Kcna2 depletion, indicating that Ube2b alleviated neuropathic pain via mediating Kcna2 via the regulation of DNMT3a. In summary, we found that Ube2b elevation ameliorated neuropathic pain through regulating Kcna2, which might offer a novel biomarker for the therapies of neuropathic pain.

## Introduction

Neuropathic pain is a chronic disease resulted from damage of somatosensory function in peripheral nervous system and central nervous system [1]. Globally, it has been estimated that approximately 7%–10% people suffer from neuropathic pain [2]. Neuropathic pain can be caused by multiple risk factors, including toxicity, surgery, and trauma [3]. Neuropathic pain seriously reduces the quality of life in patients, which is regarded as a threat to the public health worldwide [4]. Up till now, the clinic intervention and treatment of neuropathic pain still remains elusive and it is still one of the severe diseases of the world health system [5]. Thus, to deep explore the pathogenesis and identify novel biomarkers for the treatment of neuropathic pain are of huge value.

Ubiquitin proteasome system (UPS) is a fundamental mechanism that mediates protein expression and is implicated with neural development, and brain structure and functions maintenance [6,7]. UPS has been reported to induce synaptic plasticity, formation and maintenance of memory [8]. Exposure to addictive drugs leads to alterations of the expression of UPS components and increases in polyubiquitinated proteins in brain reward circuits [9]. Ubiquitin conjugating enzyme E2B (Ube2b), also known as RAD6B, is a member of the ubiquitin conjugating enzyme family. Ube2b has been identified to be a vital part of neural DNA DSBs repair. Ube2b depletion reduces H2B (histones H2B) ubiquitination, which contributes to elevate the instability of genome and neurodegeneration [9]. In the dorsal hippocampus of heroin administered rats, Ube2b modulates the DNMT3a (DNA methyltransferase 3 alpha) ubiquitination degradation and CaMKK1 gene promoter demethylation to increase the level of CaMKK1, and thus activates the CaMKK1/CaMKKα/βPIX/RAC1 cascade reaction and leads to drug-triggered actin aggregation and behavioral plasticity [10]. These studies indicate that Ube2b is essential in modulating nerve function. Moreover, an Ube2b gene promoter variant is detected in idiopathic azoospermia patients, and the Ube2b variant confers a high risk for azoospermia patients in a Chinese population [11]. Ube2b is as the downstream target of miR-455-5p, miR-455-5p promotes cell growth of oral squamous cancer cells by repressing Ube2b expression [12]. Although Ube2b is implicated with several diseases, whether Ube2b play a role in CCI-induced neuropathic pain is still elusive.We hypothesized that upregulation of Ube2b may ameliorate neuropathic pain via regulating Kcna2 in primary afferent neurons. Thus, the purpose of this study was to probe the role of Ube2b in neuropathic pain.

## Materials and methods

### Animal and chronic constriction injury (CCI) treatment

In total, 36 adult male Sprague-Dawley rats (200–230 g; Guangzhou Animal Laboratory Center) were caged with sufficient food and water at 25°C. CCI rat model was constructed by ligating the left sciatic nerve according to the previously reported with minor modification [13]. After 7 days of adaptation, rats were randomly divided into sham group, and CCI group. In CCI group, rats were anesthetized via intraperitoneal injection of chloral hydrate (300 mg/kg). The left sciatic nerve was exposed in a sterile environment, and 4 sides sciatic nerves were tied with 8–0 wires at an interval of 1 mm. In the sham group, rats were subjected to the same operation except that the nerves were not ligated. Finally, rats were scarified and the spinal cord tissues were collected at postoperative days 0, 3, 7, and 14. All animal experiments were approved by the Ethics Committee of Xinhua Hospital, School of Medicine, Shanghai Jiao Tong University for the use of animals and conducted in accordance with the National Institutes of Health Laboratory Animal Care and Use Guidelines.

### Cell culture and transfection

Mouse hippocampal neuronal cell line (HT22) were obtained from American Type Culture Collection (Manassas, USA) and grown in Dulbecco’ modified Eagle’s medium (DMEM) providing 1% streptomycin/penicillin and 10% fetal bovine serum (FBS) at 37°C with 5% CO_2_ [14,15]. Ube2b and DNMT3a overexpression vectors (Ube2b and DNMT3a) and short hairpin RNAs (shRNAs) targeting Ube2b and DNMT3a (shUbe2b and shDNMT3a) were purchased from GenePharma (Shanghai, China). These vectors were transfected into HT22 cells via Lipofectamine 2000 (Invitrogen, USA).

### Constructions and adeno-associated virus package

Adeno-associated Virus (AAVs) Ube2b and AAV-shKcna2 and their corresponding negative control AAV (AAV-NC and AAV-shNC) were purchased from Systems Biosciences (Mountain View, USA). Post 3 d surgery, AAVs were intrathecally injected into the rats for elevation of Ube2b and silencing of Kcna2 in rats.

### Quantitative real-time polymerase chain reaction (RT-qPCR)

RT-qPCR was performed to assess gene expression as previously reported [16]. RNA was isolated from spinal cord tissues utilizing TRIzol (Thermo Fisher Scientific, USA). The reverse transcription of cDNA from total RNA was subjected to a cDNA Reverse Transcription Kit (Thermo Fisher Scientific). PCR reaction was performed on SYBR Green Real-Time PCR Master Mix (Thermo Fisher Scientific) and β‑actin was served as internal reference for Ube2b. Relative level of Ube2b was calculated based on 2^–ΔΔCt^ methods. The sequences of primers used were displayed in Table 1.

**Table 1.** The primers for RT-qPCR

Gene	Forward sequence (5ʹ-3ʹ)	Reverse sequence (5ʹ-3ʹ)
Ube2b	GCTGGATGAACCGAATCCTA	TTCAACAATGGCCGAAACTC
β‑actin	AGAGATGGCCACGGCTGCTT	ATTTGCGGTGGACGATGGAG

### Hematoxylin-eosin (H&E) staining

H&E staining was used to examine the pathological changes of spinal cord tissues in rats following previously reported [17]. The spinal cord tissues slices were paraffin-treated and then dewaxed followed by rehydration with dimethylbenzene and gradient alcohol. Furthermore, the tissues sections were subjected to hematoxylin for 2 min and rinsed. Afterward, hydrochloric acid alcohol was supplemented in the sections for 2 s and washed. Then, eosin was added and incubated with the sections for 2 min. Subsequently, the slices were dried and photos were captured. Three sections of each sample in five random sights were selected to analyze the pathological changes of spinal cord tissues.

### Western blot analysis

Western blot analysis was carried out to assess protein expression as previously described [16]. Sodium dodecyl sulfate polyacrylamide was applied for isolation of proteins followed by transferring to the poly vinylidene difluoride membrane. After being sealed by milk, proteins were grown with primary antibodies overnight at 4°C. Post washing, secondary antibodies conjugated with horse radish peroxidase (Abcam, Shanghai, China) were supplemented for 1 h followed by exposure to an enhanced chemiluminescence detection system. The primary antibodies included anti-Ube2b (1/1000, ab128951, Abcam), anti-DNMT3a (1/2000, ab188470, Abcam), anti-Kcna2 (1 µg/ml, ab192758, Abcam), and anti-β-actin (1 µg/ml, ab8226, Abcam).

### Thermal hyperalgesia and mechanical allodynia measurement

Paw withdrawal thermal latency (PWTL) was applied for detection of thermal hyperalgesia to the radiant heat stimulation (Plantar Analgesia Meter; IITC Life Science, USA) following the previously described [18]. The duration from the start to paw withdrawal was recorded using a digital timer. The cutoff time was set at 20 s.

Mechanical allodynia was examined via the paw withdrawal mechanic threshold (PWMT) by Von Frey hair stimulation according to the previously described [18]. Rats were caged in a plexiglass floor with an ascending series of Von Frey hairs exposure for 5 s–6 s at 5-min intervals.

### Immunofluorescence (IF) staining

IF staining was used to analyze the levels of genes in spinal cord tissues of rats as previously described [19]. Rat spinal cord tissues were fixed by 4% paraformaldehyde and sectioned to 30 μm slices. Being blocked by phosphate buffer saline (PBS) supplemented with 5% normal goat serum and 0.3% Triton X-100, the slices were grown with sections were incubated with antibodies against Ube2b (ab277105, Abcam), DNMT3a (ab188470, Abcam), Kcna2 (ab55987, Abcam), β-actin (ab6276, Abcam), and NeuN (ab177487, Abcam) overnight at 4°C. Then, the secondary antibodies were filled in at room temperature and grown for 2 h. Three sections of each sample in 5 random sights were selected to analyze the expression of these proteins. Finally, a laser-scanning microscopy (Carl Zeiss LSM710, Germany) was employed to obtain the fluorescent images.

### Statistical analysis

Statistical analyses were implemented via SPSS17.0 statistical software. All data were represented as means ± SD. All experiments were repeated at least three times. Student’s *t*-test was applied for differences comparison between groups and One-way ANOVA was employed for analyzing the differences among multiple groups. Statistical significance was set at p < 0.05.

## Results

Neuropathic pain is a kind of pain caused by damage to somatosensory nervous system. Ube2b is validated to be implicated with nerve function, but the underlying mechanism is still unknown. Here, we determined whether Ube2b can affect neuropathic pain by regulating Kcna2. We constructed CCI rat model by ligating the left sciatic nerve, and determined the functional role of Ube2b in neuropathic pain. Then, the regulatory mechanism among Ube2b, Kcna2, and NMT3a was verified in hippocampal neuron cell line. This work revealed that Ube2b may be a novel biomarker in neuropathic pain treatment.

### Ube2b expression was decreased in CCI rats

**Figure 1.**
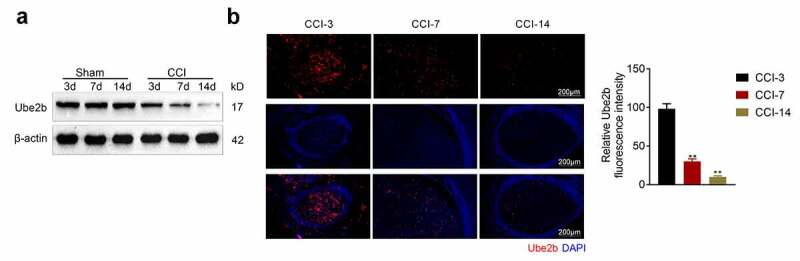
Ube2b expression was decreased in CCI rats

To evaluate the role of Ube2b in neuropathic pain, a CCI rat model was successfully constructed. As depicted in Figure 1a, the Ube2b protein level in spinal cord tissues from CCI group was evidently lower than sham group and gradually reduced on the 3^rd^, 7^th^, and 14^th^ day following operation. Moreover, the results obtained from IF staining also showed that the level of Ube2b in spinal cord tissues from CCI group was obviously decreased on the 7^th^, and 14^th^ day postoperation (Figure 1b). To sum up, Ube2b expression was decreased in spinal cord tissues of CCI rats.

CCI rat model was constructed by ligating the left sciatic nerve. Sham-operated rats were served as control. (A) Western blot analysis was performed to assess the expression of Ube2b in spinal cord tissues of rats on 3, 7, and 14 d postoperation. (B) IF staining was utilized for evaluating the level of Ube2b in spinal cord tissues of rats on 3, 7, and 14 d postoperation. N = 6. One-way ANOVA analysis. **p < 0.01 relative to CCI-3 group. CCI: chronic constriction injury; IF: immunofluorescence.

### Ube2b elevation alleviated the mechanical hyperalgesia and thermal hyperalgesia in rats

**Figure 2.**
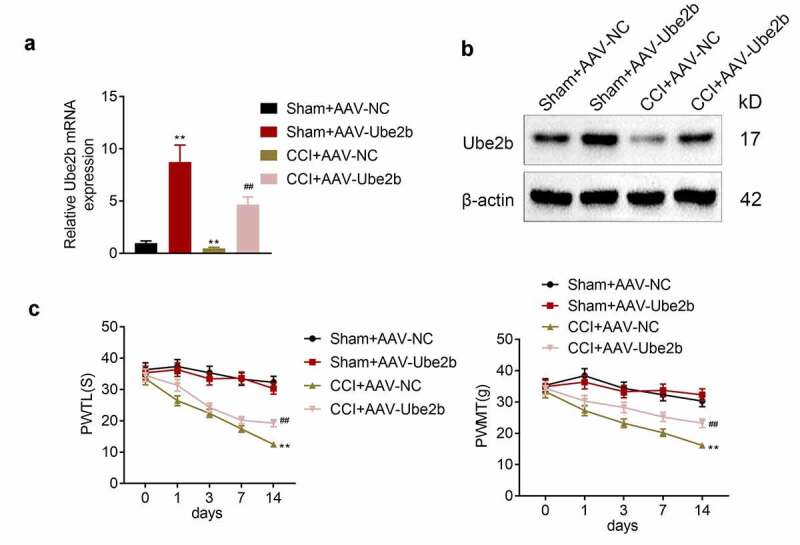
Ube2b elevation alleviated the mechanical hyperalgesia and thermal hyperalgesia in rats

Furthermore, the function of Ube2b in neuropathic pain was measured. Firstly, Ube2b was elevated in spinal cord tissues of rats in both CCI and sham groups via AAV transfection. The data delineated that the Ube2b mRNA and protein levels were dramatically increased in Sham + AAV-Ube2b group as compared with Sham + AAV-NC group. In addition, the Ube2b mRNA and protein levels were also apparently higher in CCI + AAV-Ube2b group than that in CCI + AAV-NC group (Figure 2a-b). Afterward, we observed that CCI rats displayed a significant decrease of PWTL and PWMT with respect to sham-operated rats. Both the PWTL and PWMT were elevated due to the upregulation of Ube2b in CCI rats (Figure 2c), suggesting that Ube2b elevation alleviated the mechanical hyperalgesia and thermal hyperalgesia in rats. In a word, Ube2b overexpression ameliorated neuropathic pain on the 14^th^ day after model induction.

CCI rat model was constructed by ligating the left sciatic nerve. CCI rats and Sham-operated rats were injected with AAV-Ube2b or AAV-NC. The expression of Ube2b in spinal cord tissues of rats was measured via RT-qPCR (A) and Western blot analysis (B). (C) The thermal hyperalgesia and mechanical allodynia of rats were examined via PWTL and PWMT. N = 6. One-way ANOVA analysis. **p < 0.01 relative to Sham + AAV-NC group, ^##^p < 0.01 relative to CCI+ AAV-NC group. CCI: chronic constriction injury; RT-qPCR: quantitative real-time polymerase chain reaction; PWTL: paw withdrawal thermal latency; PWMT: paw withdrawal mechanic threshold.

### Ube2b elevation suppressed chronic sciatic nerve injury

**Figure 3.**
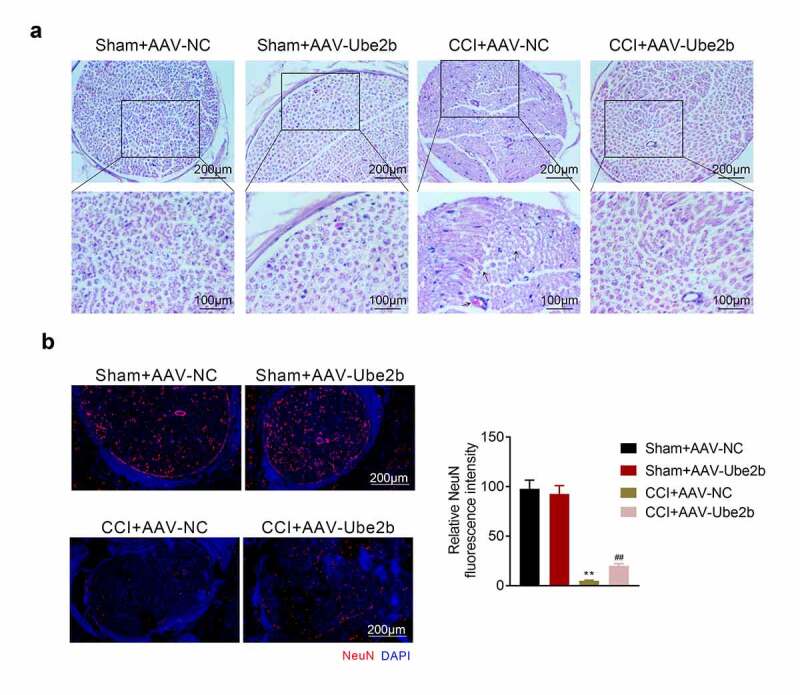
Ube2b elevation suppressed chronic sciatic nerve injury

Thereafter, whether Ube2b modulated chronic sciatic nerve injury was probed. Based on the results from H&E staining, the sham rats displayed a normal histological structure of spinal cord tissues, and the spinal fibers of the sciatic nerve were closely arranged. However, the nerve fibers were obviously swollen, the number of neurons was decreased, and the capillaries dilated and congested in CCI + AAV-NC group. Elevation of Ube2b alleviated these damage of spinal cord tissues in CCI rats (Figure 3a). Besides, the index of neurons (NeuN) was tested via IF staining, the data revealed that the level of NeuN was distinctly declined due to CCI treatment as compared with sham group. The level of NeuN was increased in CCI rats following administration of AAV-Ube2b (Figure 3b). Taken together, Ube2b elevation suppressed chronic sciatic nerve injury in rats.

CCI rat model was constructed by ligating the left sciatic nerve. CCI rats and Sham-operated rats were injected with AAV-Ube2b or AAV-NC. (A) H&E staining was used to examine the pathological changes of spinal cord tissues in rats. (B) The level of NeuN in spinal cord tissues of rats was exhibited by IF staining. N = 6. One-way ANOVA analysis. **p < 0.01 relative to Sham + AAV-NC group, ^##^p < 0.01 relative to CCI+ AAV-NC group. CCI: chronic constriction injury; H&E: Hematoxylin-eosin; IF: immunofluorescence.

### Ube2b regulated the expression of DNMT3a and Kcna2

**Figure 4.**
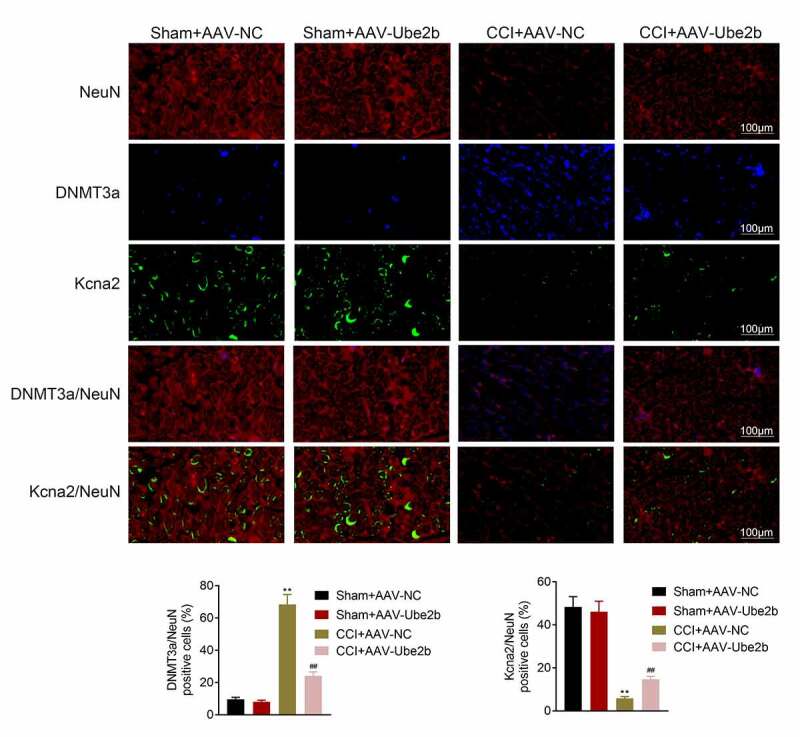
Ube2b regulated the expression of DNMT3a and Kcna2

Next, the regulatory mechanisms underlying Ube2b in neuropathic pain were investigated. As displayed in Figure 4, DNMT3a was mainly distributed in cell nucleus while Kcna2 was largely located in cytoplasm. Ube2b elevation had no evident change in the levels of DNMT3a and Kcna2 in sham group. The level of DNMT3a was increased, while the level of Kcna2 was decreased in CCI rats. However, these effects conferred by CCI treatment in rats were offset on account of Ube2b enhancement. To conclude, Ube2b regulated the expression of DNMT3a and Kcna2.

CCI rat model was constructed by ligating the left sciatic nerve. CCI rats and Sham-operated rats were injected with AAV-Ube2b or AAV-NC. The levels of DNMT3a and Kcna2 in spinal cord tissues of rats were displayed by IF staining. N = 6. One-way ANOVA analysis. **p < 0.01 relative to Sham + AAV-NC group, ^##^p < 0.01 relative to CCI+ AAV-NC group. CCI: chronic constriction injury; IF: immunofluorescence.

### Ube2b promoted the level of Kcna2 via regulating DNMT3a

**Figure 5.**
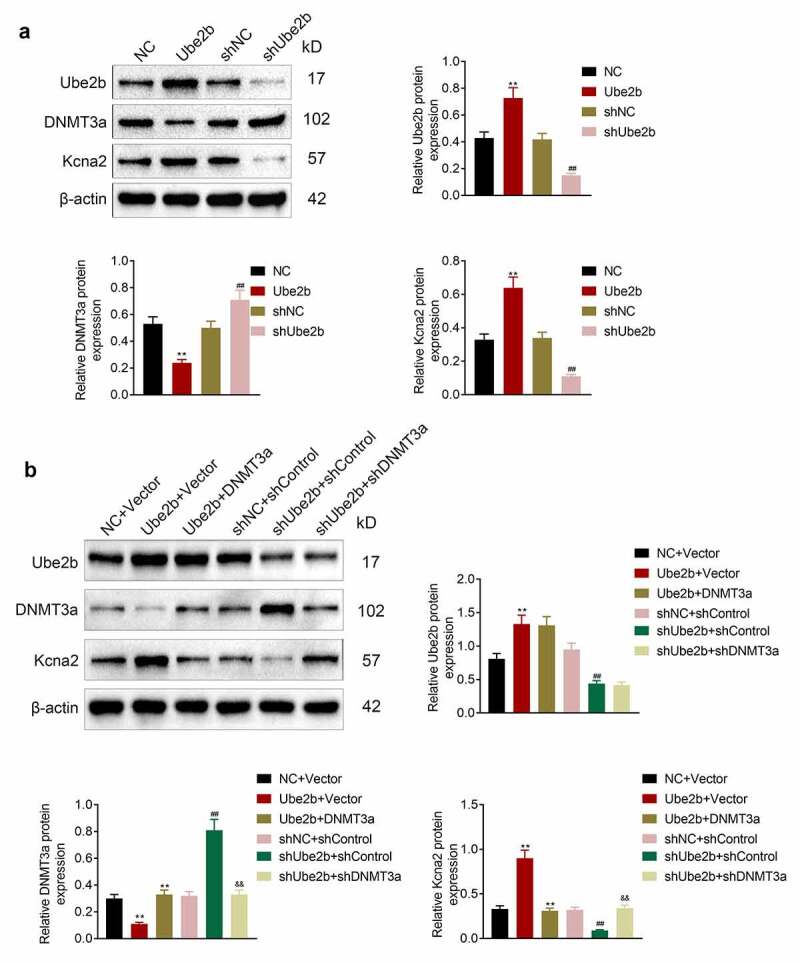
Ube2b promoted the level of Kcna2 via regulating DNMT3a

Afterward, whether Ube2b can mediate Kcna2 via regulating DNMT3a was investigated in hippocampal neuronal cell line HT22. As depicted in Figure 5a, Ube2b elevation decreased the level of DNMT3 but increased the expression of Ube2b and Kcna2 in HT22 cells. Ube2b silencing enhanced the expression of DNMT3a but reduced the level of Ube2b and Kcna2 in HT22 cells. Besides, Ube2b overexpression-mediated downregulation of DNMT3 and upregulation of Ube2b and Kcna2 was reversed by DNMT3 enhancement. However, DNMT3 reduction offset Ube2b deficiency-modulated DNMT3 enhancement and Kcna2 decline in HT22 cells (Figure 5b). In summary, Ube2b promoted the level of Kcna2 via regulating DNMT3a.

(A) HT22 cells were transfected with Ube2b, NC, shKcna2 or shNC. Western blot analysis was performed to identify the protein levels of Ube2b, DNMT3a, and Kcna2 in the HT22 cells. (B) HT22 cells were transfected with Ube2b, NC, DNMT3a, Vector, shUbe2b, shNC, shDNMT3a, shControl. Western blot analysis was performed to identify the protein levels of Ube2b, DNMT3a, and Kcna2 in the HT22 cells. N = 3. One-way ANOVA analysis. **p < 0.01 relative to NC + Vector group, ^##^p < 0.01 relative to shNC + shControl group, ^&&^p < 0.01 relative to shUbe2b + shControl group.

### Ube2b modulates neuropathic pain via mediating Kcna2

**Figure 6.**
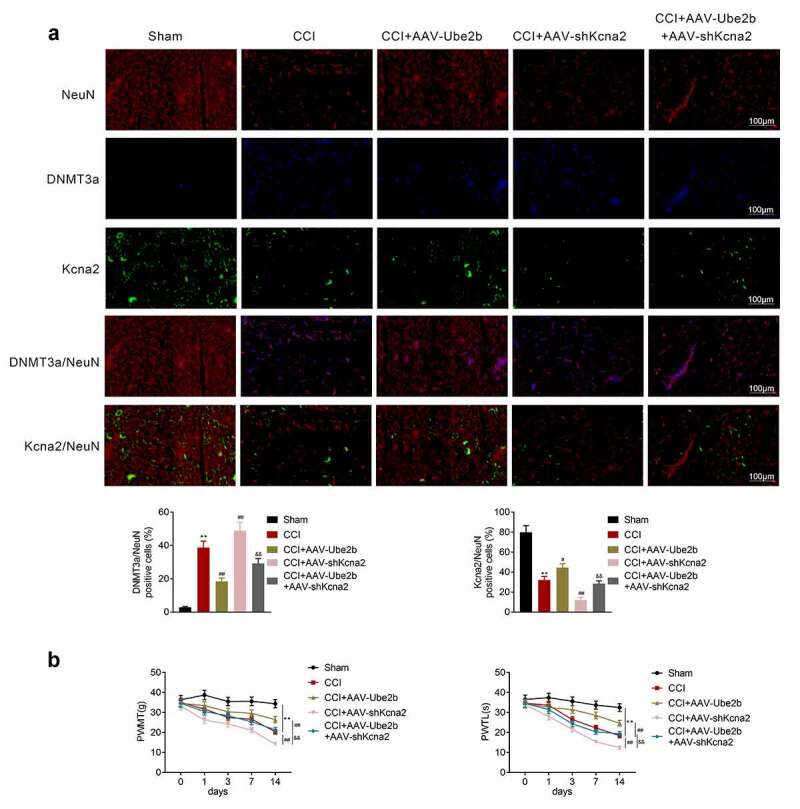
Ube2b modulates neuropathic pain via mediating Kcna2

Finally, we explored whether Ube2b can exert its function in CCI rats via mediating Kcna2 expression. According to the results of IF staining, Ube2b upregulation reduced DNMT3a expression and enhanced Kcna2 expression in CCI rats. Kcna2 downregulation counteracted Ube2b upregulation induced the decrease of DNMT3a and increase of Kcna2 (Figure 6a). The mechanical hyperalgesia and thermal hyperalgesia were decreased in CCI rats, which was rescued by Ube2b upregulation. Additionally, Ube2b overexpression-modulated increase of mechanical hyperalgesia and thermal hyperalgesia were reversed by Kcna2 depletion (Figure 6b). All in all, Ube2b modulated neuropathic pain via mediating Kcna2.

CCI rat model was constructed by ligating the left sciatic nerve, followed by injection of AAV-Ube2b, AAV-shKcna2 or AAV-Ube2b combined with AAV-shKcna2. Sham-operated rats were served as control. (A) IF staining was used for analyzing the levels of Ube2b, DNMT3a and Kcna2 in spinal cord tissues of rats. (B) The thermal hyperalgesia and mechanical allodynia of rats were examined via PWTL and PWMT. N = 6. One-way ANOVA analysis. **p < 0.01 relative to sham group, ^##^p < 0.01 relative to CCI group, ^&&^p < 0.01 relative to CCI + AAV-Ube2b group. CCI: chronic constriction injury; IF: immunofluorescence; PWTL: paw withdrawal thermal latency; PWMT: paw withdrawal mechanic threshold.

## Discussion

Neuropathic pain is a kind of pain caused by injury to somatosensory nervous system, which disturbs the quality of life of patients [20]. Currently, neuropathic pain, as a chronic pain relative to other kinds of pain, is still a medical problem for clinicians [21]. To uncover more reliable biomarkers for neuropathic pain treatment is of vital importance. Previously, multiple researchers have provided evidence that message RNAs (mRNAs) could code proteins to modulate the development of neuropathic pain. For instance, BDNF epigenetic modification regulates neuropathic pain in CCI rat model through regulating miR-30a-3p and EP300 expression [22]. Akt3 mediates neuropathic pain via acting as a target of miR-20b-5p in CCI rats [23]. CCI-induced neuropathic pain is aggravated by EZH2 in a rat model [24]. According to bioinformatic analysis and validation of experiments, Slc6a19os and SOX11 are two vital regulators of neuropathic pain [25]. Ube2b is validated to be implicated with several diseases. For instance, high expression of UBE2B is closely associated with poor response and low survival rate in rectal cancer patients following chemoradiotherapy [26]. UBE2B participates in myofibrillar protein loss in catabolic C2C12 myotubes, which contributes to maintain muscle protein homeostasis during catabolic conditions [27]. However, the role of Ube2b in neuropathic pain still requires to be elucidated. Herein, Ube2b expression was confirmed to be decreased in CCI rats. We speculated that Ube2b expression may be decreased with the progress of neuropathic pain. Moreover, Ube2b elevation alleviated the mechanical hyperalgesia and thermal hyperalgesia in CCI rats. In addition, Ube2b elevation suppressed chronic sciatic nerve injury. On the whole, Ube2b could ameliorate the development of neuropathic pain.

Kcna2 (also named Kv1.2) is the subunit of the voltage-gated potassium channel family [28,29]. Kcna2 modulates the excitability of neurons by regulating resting membrane potential and repolarization post action potentials [30]. Kcna2 dysfunction is involved in the pathogenic mechanism of neuropathic pain [31]. Kcna2 restrains nerve injury-triggered neuropathic pain via acting as a target of miR-137 [32]. In addition, Kcna2 expression is associated with hyperalgesia of osteoarthritis pain [33]. Nonetheless, whether Kcna2 exerted a role in neuropathic pain is elusive.

In recent years, the role of epigenetic modification including DNA methylation in the pathogenesis of diseases has been widely proposed by researchers [34]. DNA methylation modification genes such as DNMT1 and DNMT3a are frequently identified to be implicated with various diseases. For instance, DNMT1 and DNMT3a upregulation aggravates the hypermethylation of antitumor genes in human pituitary adenomas [35]. DNMT1 modulates interferon regulatory factor 8 expression via DNA methylation to regulate cisplatin-induced kidney injury [36]. Tgfb1 promoter is mediated by DNA demethylation in mesangial cells to regulate diabetic kidneys [37]. More importantly, DNA methyltransferases DNMT1 and DNMT3a alleviates Kcna2 expression in primary afferent neurons to accelerate neuropathic pain [31,33]. Increased endogenous Kcna2 antisense promotes neuropathic pain symptoms by reducing the expression of Kcna2 in primary afferent neurons [38]. Hence, KCNA2 might be a pivotal regulator of neuropathic pain via DNA methylation modification. In our work, we corroborated that Ube2b upregulation elevated the level of Kcna2 via suppressing DNMT3a, implying that Ube2b modulated Kcna2 expression through inhibiting the DNMT3a-mediated DNA methylation. Rescue assays unveiled that Ube2b alleviated neuropathic pain via mediating Kcna2 through DNMT3a pathway.

The study of De et al. has demonstrated that Human Schwann cells express ADH/TRPA1/NOX1 to chronic neuroinflammation and allodynia, and TRPA1 antagonists may attenuate some symptoms of alcohol-related pain [39–41]. HCAR2 is highly expressed in the sciatic nerve and the dorsal root ganglia in neuropathic mice, and HCAR2 participates in regulating neuropathic pain [42]. Additionally, a previously study has confirmed that dendritic cells in tumor microenvironment sensitize nociceptor sensory neurons via paracrine inflammatory and growth factors, which contributes to promote neuropathic pain [43]. Long noncoding RNA NEAT1 elevates MAPK1 expression by acting as sponge of miR-211-5p, and thus accelerates inflammatory response of spinal cord injury [44]. These data confirms that inflammatory response plays a crucial role in neuropathic pain. This work revealed that Ube2b promoted the level of Kcna2 via regulating DNMT3a, thereby ameliorating neuropathic pain. Whether Ube2b can affect neuropathic pain by regulating inflammatory response still needs further research. We will conduct research on this topic.

## Conclusion

In summary, the current work demonstrated that Ube2b elevation ameliorated neuropathic pain through regulating Kcna2. Thus, this work might offer a novel biomarker for the therapies of neuropathic pain.

## Highlight

1. Ube2b expression was decreased in CCI rats.
2. Ube2b elevation alleviated the mechanical hyperalgesia and thermal hyperalgesia in rats.
3. Ube2b elevation suppressed chronic sciatic nerve injury.
4. Ube2b regulated the expression of DNMT3a and Kcna2.
5. Ube2b promoted the level of Kcna2 via regulating DNMT3a.
6. Ube2b modulated neuropathic pain via mediating Kcna2.
